# A needs assessment of people living with diabetes and diabetic retinopathy

**DOI:** 10.1186/s13104-016-1870-4

**Published:** 2016-02-01

**Authors:** Claudette E. Hall, Anthony B. Hall, Gerjo Kok, Joyse Mallya, Paul Courtright

**Affiliations:** Department of Work and Social Psychology, Faculty of Psychology and Neuroscience, University Maastricht, P.O. Box 616, 6200 MD Maastricht, The Netherlands; Newcastle Eye Hospital Research Foundation, 182 Christo Road, Waratah, NSW 2289 Australia; Kilimanjaro Christian Medical Centre, P.O. Box 3010, Moshi, Tanzania; KCCO International, Division of Ophthalmology, H53 OMB, Groote Schuur Hospital, University of Cape Town, Observatory, 7925 South Africa

**Keywords:** Diabetic retinopathy, Screening, Self-efficacy, Social stigma, Diabetes mellitus, Diabetes complications, Needs assessment, Evidence based healthcare

## Abstract

**Background:**

The Kilimanjaro Diabetic Programme was initiated in response to the needs of people living with diabetes (PWLD) to identify barriers to uptake of screening for diabetic retinopathy, to improve management of diabetes, and establish an affordable, sustainable eye screening and treatment programme for diabetic retinopathy. Intervention Mapping was used as the framework for the needs assessment.

**Methods:**

A mixed methods approach was used. Five psychometric measures, Diabetes Knowledge Questionnaire, Diabetes Health Beliefs, Self-Efficacy scale, Problem Areas in Diabetes scale, and Hopkins Scale Checklist-25 and a structured interview relating to self-efficacy, addressing disclosure of living with diabetes and life-style changes were used to triangulate the quantitative findings. These were administered to 26 PWLD presenting to rural district hospitals.

**Results:**

The interviewees demonstrated low levels of perceived stigma regarding disclosure of living with diabetes and high levels of self-efficacy in raising community awareness of diabetes, seeking on going treatment from Western medicine over traditional healers and in seeking care on sick days. Self-efficacy was high for adjusting diet, although comprehensive dietary knowledge was poor. Negative emotions expressed at diagnosis, changes in life style and altered quality of life were reflected in high levels of anxiety and depression.

**Conclusions:**

Low levels of stigma surrounding living with diabetes were linked to a desire to raise community awareness of diabetes, help others live with diabetes and to secure social support to access hospital services. Confusion over what constituted a healthy diet showed the importance of comprehensive, accessible diabetes education, essential to ensuring good glycaemic control, and preventing diabetic complications, including diabetic retinopathy. Low levels of self-efficacy along with high levels of anxiety and depression may have a negative impact on the uptake of screening for Diabetic Retinopathy. The findings of this needs assessment led to the planning and delivery of a comprehensive health intervention programme for PLWD in Kilimanjaro Region. The programme has provided them with support, resources, education, and screening for diabetic retinopathy at the regional hospital and at district level with mobile digital retinal cameras, an electronic diabetic database and computerised follow up to ensure continuity of care.

## Background

Diabetes has reached epidemic proportions with an estimated 285 million people living with diabetes globally in 2010 and 366 million in 2011 [1]. Global figures for those living with the disease must be viewed with caution due to difficulties in areas where social, economic and political factors make regular diabetes services difficult [1, 2]. It is estimated that in 2010 approximately 12.1 million people in Sub-Saharan Africa (SSA) were living with diabetes.

Diabetes in Africa is perceived to be more common amongst affluent urban dwellers than among poorer members of communities living in rural areas [3]. This urban versus rural pattern appears to be shifting in Africa, with an increase in diabetes among countries that are of low and middle income, and amongst the poorer of these nations. In 2010 in Tanzania the prevalence of diabetes was estimated at 5.8 % in urban and 1.7 % in rural areas and in the next two decades there is a projected 50 % increase in the prevalence of diabetes [3].

The relationship between diabetes and blindness due to diabetic retinopathy (DR) is well established. Global estimates for the prevalence of blindness are 0.55 %, of which blindness due to DR ranges from 1 to 4.8 % of this total [4]. In Sub-Saharan Africa it is estimated that 21–25 % of people with type 2 diabetes have diabetic retinopathy (DR) at diagnosis [5].

Screening people living with diabetes (PLWD) for DR meets the World Health Organisation criteria for screening and blindness prevention [6]. Screening for DR is effective if individuals are identified and DR treated early, before loss of visual acuity occurs. The longer the duration of diabetes the greater the risk to the individual of micro-vascular complications including DR, neuropathy and nephropathy [7].

When the study was conceived there were no up to date data available on the incidence and prevalence of diabetes and of DR for the Kilimanjaro Region [8]. When the diabetic clinic was first established at Kilimanjaro Christian Medical Centre (KCMC) hospital in 1996 a study of 474 patients found a prevalence of 14.5 % among this group [8].

There are no current data on uptake of diabetic services, how effectively health care workers communicate health information to PLWD; and in particular what information PLWD receive about eye health. Neither were there documented experiences or guidance concerning the establishment of DR screening services in developing countries.

A study conducted at KCMC Hospital in 2006 showed that a number of issues need to be addressed if blindness due to DR is to be prevented in the Kilimanjaro Region. The study found that in spite of specialist eye services at KCMC Hospital to support the diabetic clinic, most patients presented late for treatment of DR. As part of this study, a nurse counsellor attended the twice weekly adult diabetic clinic to give PLWD information about DR, encouraged them to ask questions and gave them a free referral to the eye clinic that could be used at their convenience. The study demonstrated that free referral for DR screening from the KCMC Diabetic Clinic to the nearby (less than 100 meters) KCMC eye clinic only managed to increase uptake to by 36 %. In this diabetic population 93, 71 % had not had a dilated eye examination in the past year. The authors suggested that knowledge, by itself, does not result in a change in health behaviour [9].

We aimed to develop a health promotion programme that would be informed and supported by the local community, draw upon local resources and be evidence based. Using intervention mapping [10] as the framework for the Kilimanjaro Diabetic Programme (KDP) [11, 12] we began by conducting a needs assessment (NA) [10]. A needs assessment is the first of 6 steps in planning health promotion programmes using an intervention mapping approach [10]. Bartholomew describes how this uses the PRECEDE model as a conceptual framework. The model examines a number of determinants including environmental and behavioural factors which have an effect on the health problems and ultimately the quality of life of the population at risk. The aim of this study was to provide an overview of the needs of a self-selecting group of people known to be living with diabetes in the Kilimanjaro Region.

### Behavioural factors

A comprehensive ecological approach is valuable in gaining an understanding of the eye health behaviour of PLWD and in identifying barriers to the uptake of eye screening services, in order to establish an affordable, sustainable eye screening and treatment programme for DR [10]. This paper describes the first part of this process. The following concepts form the theoretical basis for this study.

Until the early 1990s knowledge, attitudes and belief models were the focus of diabetes education [13]. Subsequently it was recognised that PLWD required education from the perspective of self-management with an emphasis on self-empowerment and self-efficacy. This allows PLWD to manage their self-care in order to live a healthy life and prevent potential micro-vascular and macro-vascular complications, such as, DR [14].

Bandura [15] describes ‘perceived self-efficacy’ as beliefs in an individual’s capabilities to plan, organize and conduct behaviour to achieve set goals according to self-set standards. It is not enough just to know what to do. The individual must have an effective and organised set of sub-skills; cognitive, social, emotional and behavioural, that can be engaged appropriately under different and sometimes difficult and challenging situations [15]. If an individual has a strong perceived self-efficacy they will set high goals, be firmly committed to achieving these goals and be able to persist with considerable effort in the face of difficulties in achieving their goals. The converse is true [16, 17].

#### Diabetes knowledge

What is the role of education in self-management of diabetes? Certainly individuals require knowledge and skills in all aspects of their treatment and how to prevent complications in order to be effective in self-care. However, whilst knowledge and skills are important they do not of themselves ensure competent self-care or behaviour change [10]. Education and skills training of PLWD is an essential starting point to tailor diabetes education. So too is assessment of knowledge, to address knowledge gaps and provide appropriate and adequate training. This is a challenge for health intervention programme planners.

#### Anxiety and depression

Living with a chronic disease, such as diabetes is known to lead to psychosocial distress and depression [18–21]. The work done by Lee et al. [22] reports that researchers in Eastern Africa in general and specifically in Tanzania have found that in primary care, mental health tends to be undiagnosed. In East Africa symptoms of depression are usually dismissed as being psychological or due to social problems. In Tanzania depression is thought to be due to psychosocial problems [23]. It is clear from the work of these researchers that more needs to be done to develop appropriate cultural scales that reflect both indigenous classifications and expressions of illness [24].

#### Social stigma

Social stigma is complex and relevant to our NA of PLWD. Stigma has played a historical role from the earliest of times when bells were rung to warn of the approach of lepers [25]. Social stigma is experienced as prejudice and negative stereotyping for a number of reasons, including for example, physical illness, disfiguration or deformity, personality traits perceived as deviating from social or cultural norms as in mental illness, drug or alcohol addiction, sexual orientation, and tribal taboos, i.e. ethnicity, race and religion [26, 27]. People who are stigmatised by their community are ostracised, devalued and kept at a distance [25]. Diabetes is an invisible illness, so PLWD may choose either to conceal their illness and appear ‘normal’ to others or reveal it and face possible stigmatization by those who are well. Diabetes may be regarded as a self-imposed illness, due to a lack of self-control and laziness. We wanted to investigate the concept that there is stigma towards those with diabetes in our NA of Tanzanian PLWD.

#### Assessment tools

There are a number of tools that have been developed to study these issues. Some have been developed for use in PLWD and we therefore chose and adapted these rather than more general quality of life or vision questionnaires. The following five psychometric measures were used to evaluate the needs of the group of PLWD in the study; Self-efficacy scale [28], Diabetes Health Belief [29], Diabetes Knowledge Questionnaire [30], Problem Areas In Diabetes [31] and Hopkins Scale Checklist-25 [22, 32].

*Self*-*Efficacy* (SE) Measuring SE activities for PLWD involves questions on carrying out tasks that focus on treating their disease and preventing complications. From these tasks three distinct classes of activities can be identified on a type two diabetes SE scale: [28]

1. Performing activities that relate to the treatment of diabetes: taking medication, oral or injection; maintaining a healthy diet and physical exercise.
2. Self-observation: monitoring and control of blood glucose, body weight, condition of feet and general health.
3. Other self-regulation activities: recognition and correction of hypoglycaemia and hyperglycaemia, variety in diet, preparing to travel away from home, self-regulation in recognition of weight gain and appropriate response to illness and stressors.

The SE scale for people living with type two diabetes was developed to measures both situation specific tasks and whether people consider themselves capable of completing them [28].

*Diabetes Health Beliefs* (DHB), Health beliefs have been shown to have a direct and indirect effect on metabolic control. Use of this 25 item DHB measure focuses on self-management and measures health outcomes [33].

*The Diabetes Knowledge Questionnaire* (DKQ) was developed for individuals who are not first language English speakers, with a low level of literacy, to test the outcome of diabetes self-management education [30].

*Problem Areas in Diabetes* (PAID) is a 20 item psychometric measure of the emotional distress of living with a chronic disease and the burden of self-management from the perspective of the individual living with diabetes [31].

*The Hopkins Scale Checklist*-*25* (HSC-25) has been used since the 1950s in a variety of settings to measure levels of anxiety and depression among individuals in a wide range of populations and of diverse cultural origins. This psychometric measure has more recently been adapted for use among Kiswahili speakers within the primary care setting in Tanzania [22, 32].

#### *Eye Health Questionnaire (EHQ*)

The EHQ included questions relating to past eye health behaviour, whether individuals knew they needed to have annual eye screening and what barriers prevented them from attending for DR screening (see Table 1).

**Table 1:** Summary of Eye Health Questionnaire

Patient groups	Knowledge that DM damages eyes	Duration of DM in years	Not previously screened for DR	Accepted screening for DR	Knew about KCMC Hospital
Totals	56/79 (70.9 %)	8.69	55/79 (69.6 %)	71/79 (89.9 %)	43/79 (54.4 %)

The research question forming this needs assessment was, ‘What are the needs of people living with diabetes and diabetic retinopathy in Kilimanjaro Region?’

## Methods

The setting for this study was in 5 rural district hospitals in Kilimanjaro Region, Tanzania. Kilimanjaro Region has a population of 1,376,702 (Population census, 2002) [34]. Most roads in the area are gravel and access to health care is made either by foot, bicycle or public transport. In the rainy season some of these roads are difficult to access.

A mixed methods approach was used with one qualitative standardised questionnaire and five quantitative psychometric measures to triangulate the findings. In addition to this an eye health questionnaire (EHQ) was asked of all participants and screening for DR was offered to all who took part in the study. All PLWD who attended the health facilities where the NA were conducted were also offered screening for DR.

PLWD were invited to attend DR screening at the district hospitals with invitations being given through Faith Based Organizations, community groups and health services. The first people to arrive on each DR screening day were asked to take part in the NA interviews. Subjects completed all 7 questionnaires on the same day. These data were gathered using convenience sampling of 27 self-selecting known diabetics. All the PLWD who came to the NA day were offered screening for DR. The data from one person was discarded as it was incomplete. People under the age of 18 years and people who were considered to be too ill to take part in the study (e.g. with a high random blood sugar) were excluded from the study. People were read information about the purpose of the study gave written consent to take part in the study. Ethical approval was granted by the Ethics Committee of Kilimanjaro Christian Medical College, Tumaini University.

Socio-demographic information included age, gender, number of children, level of education, and occupation [35]. Visual acuity was calculated with reference to the International Classification of Diseases (ICD-10th revision) [36].

*Adaptation and Translation of questionnaires*—Adaptation and translation of the questionnaires from English into Swahili was conducted prior to the pilot study. The questionnaires were translated backwards and forwards by experienced staff and reviewed by the KDP working committee. Research assistants collected the psychosocial information using face-to-face administration, as some study participants were illiterate. The psychosocial instruments were marked with faces (–) to help participants with identifying the meaning of the scales on each instrument. The questions were read out in Kiswahili (the official language of Tanzania).

### Qualitative measures

A series of 21 item structured interviews were conducted, in Kiswahili, with each of the 26 participants of the NA by an experienced community health care worker (see Table 2). The interview sessions were discussed on conclusion by the interviewer (JM) and researchers (CH and AH). Each interview was taped and then transcribed and translated into English. The interviews were thematically coded using Atlas-ti 6, to establish the expressed needs of each PLWD. The data were coded into nine themes identified during the coding and re-coding process and measured in terms of self-efficacy, i.e. seeking initial diagnosis; expression of emotion at diagnosis; dialoguing with others about having diabetes (stigma); social support and raising public awareness; seeking on going treatment, engaging in dialogue about treatment; coping with sick days; seeking alternative therapy (herbalists), burden of disease and associated changes in life-style (understanding healthy living—diet and exercise). There was discussion between researchers (CH and AH) during the coding, re-coding and analysis of the structured interviews.

**Table 2:**
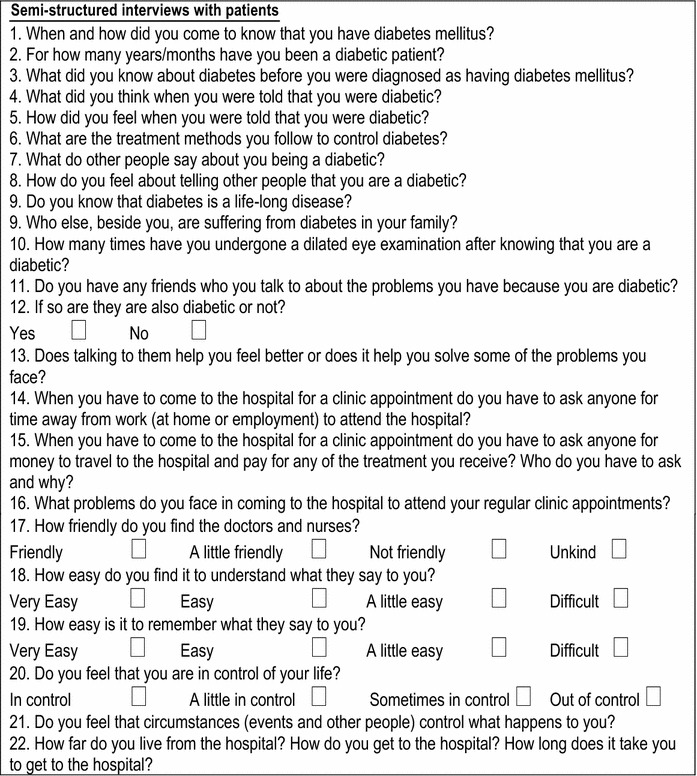
Qualitative interview questions

### Quantitative measures

The five psychometric measures that were selected to evaluate the needs of the group of PLWD in the study were administered by the interviewers in Swahili

*Self*-*Efficacy* (SE) The 20 items on 5 subscales were scored on a five point Likert scale ranging from ‘For sure not’ (1) to ‘Yes for sure’ (5). The original self-efficacy instrument for type 2 diabetes had only 4 subscales. —In the Kiswahili version the item ‘I think that I am able to keep to my diet whenever I am away from home,’ was considered to be a repetition of the item, ‘I think that I am able to keep to my diet when I will be away for a holiday or family visit’. As there are high levels of hypertension among PLWD in Kilimanjaro Region the item, ‘I think that I am able to keep my blood pressure under control’ was added as a fifth subscale. High means scores indicate high levels of SE and low mean scores indicate low levels of SE (see Table 3).

**Table 3:** Results of Self Efficacy scale

	*Self*-*efficacy* (n = 26) *m* = 4,0, STD 12.9	
	*Blood sugar* (3 items - 1, 2, 3) *m* = 3.7, STD 6.8, range 1–5	
1.	I think that I’m able to control my blood sugar myself whenever this is necessary	3.5
2.	I think that I’m able to lower my blood sugar whenever it is too high	3.6
3.	I think that I’m able to raise my blood sugar if it is too low	3.9
	*Physical exercise* (3 items—8, 11, 12) *m* = 4.5, STD 1.0, range 1–5	
8.	I think that I’m able to stay physically active enough through for instance walking or biking	4.6
11.	I think that I am able to be more physically active if this is advised by my nurse/doctor	4.6
12.	I think that I am able to change my diet in the case of increased physical activity	4.5
	*Nutrition/weight* (4 items—6,13,14,15) *m* = 3.5 STD 7.1, range 1–5	
6.	I think that I’m able to keep my weight under control	3.7
13.	I think that I am able to keep to my diet whenever I am away from home	3.2
14.	I think that I am able to keep to my diet when I will be away for a holiday or family visit	3.4
15.	I think that I am able to keep to my diet when I am having a party	3.8
	*Nutrition/treatment* (9 items—4, 5, 7, 9, 10, 16, 17, 19, 20) *m* = 4.2, STD 13.4, range 1–5	
4.	I think that I’m able to choose the right food for my diabetes	4.0
5.	I think that I’m able to keep my food varied enough	4.0
7.	I think that I’m able to check my feet and on wounds, even the smallest ones	4.4
9.	I think that I’m able to adapt my food myself if I am ill	4.3
10.	I think that I’m able to keep to my diet most of the time	3.3
16.	I think that I am able to keep to my diet if I am stressed or tense	3.6
17.	I think that I am able to consult my nurse/doctor on a yearly basis for my diabetes	4.7
19.	I think that I am able to take my medication according to the doctor’s prescription when I am away from home	5.0
20.	I think that I am able to change my medication if I am ill	4.2
	*Blood pressure* (1 items—18) *m* = 3.9, STD 1.5, range = 1–5	
18.	I think that I am able to keep my blood pressure under control	3.9

*Diabetes Health Beliefs* (DHB) [29, 33] The 25 item DHB measure focuses on self-management and measures health outcomes with 5 themes, as follows;

- One item of perceived control of diabetes.
- Total Barriers (to diet and medication are combined) with higher scores indicating higher perceived barriers.
- Social support for diet, with higher scores indicating higher perceived social support.
- Impact of job on therapy, with higher scores indicating higher perceived impact of job on diabetes therapy.
- Benefits of therapy, with higher scores indicating higher perceived benefits of therapy (see Table 4).

**Table 4:** Results of Diabetic Health Beliefs

*Diabetic Health Beliefs* (n = 26) *m* = 3.5 SD = 22.9 (n = 26)	
*Control (1) m* = 4.2, SD 0.9, range 1–5	
1. My diabetes is well controlled	4.2
*Barriers (5) m* = 3.6, SD 0.7, range 1–5	
2. I would have to change too many habits to follow my diet (diabetic foods)	3.9
3. It has been difficult following the diet (diabetic foods) the doctor ordered for me	2.5
4. I am confused by all the medication the doctor has given me	2.2
5. I would have to change too many habits to take my medication	2.9
6. Taking my medication interferes with my normal daily activities	2.4
*Social support (3)* *m* = 2.4, SD 0.4, range 1–5	
7. I have others around me who remind me to eat the right foods	3.1
8. I can count on my family when I need help following my diet (diabetic foods)	3.7
9. My husband/wife helps me follow my diet (diabetic foods)	3.9
*Impact of Job (5) m* = 2.4, SD 0.4 range 1–5	
10. If I changed “jobs” it would be easier to follow my diet (diabetic foods)	1.9
11. My work makes me so tired that it’s hard to follow my diet (diabetic foods)	2.2
12. I could control my weight if the pressures of my job weren’t so great	2.7
13. If I changed “jobs,” it would be easier to take my medication	2.4
14. I worry so much about my job that I can’t take my medication	2.0
*Benefits (11) m* = 4.3, SD 0.4, range 2–5	
15. I believe that my diet (diabetic foods) will help prevent diseases (complications) related to diabetes	4.4
16. Following a prescribed diet (diabetic foods) is something a person must do no matter `how hard’ it is	4.3
17. 1 believe that my diet (diabetic foods) will control my diabetes	4.5
18. I must follow my diet (diabetic foods) even if I don’t think I am getting better	4.2
19. In general, I believe that my diet (diabetic foods) for diabetes will help me to feel better*	4.3
20. Controlling weight is something one must do no matter how hard it is	4.2
21. I believe that my medication will help prevent diseases (complications) related to Diabetes	4.1
22. Taking medication is something one must do no matter how hard it is	4.3
23. I believe that my medication will control my diabetes	4.5
24. I must take my diabetes medication even if I don’t think I am getting better	4.3
25. I believe that my medication for diabetes will help me to feel better	4.3

A five point Likert scale was used ranging from ‘Strongly disagree’ (1) to ‘Strongly Agree’ (5).

*The Diabetes Knowledge Questionnaire* We used this 24 item DKQ [30] to broadly assess diabetes knowledge of diabetes to establish a baseline for future education needs of PLWD. There were eleven items 2, 5, 6, 8, 11, 14, 15, 16, 18, 19, 20, items rated as true. The remainder of the items, items 1, 3, 4, 7, 9, 10, 12, 23, 17, were rated as false (scoring = yes, no, or I don’t know) (see Table 5).

**Table 5:** Results of Diabetes Knowledge Questionnaire

*DKQ* (n = 26) *m* 15.9 *SD* 7.59 (n = 26)	True	False
1. Eating too much sugar and other sweet foods is a cause of diabetes	17	*9*
2. The usual cause of diabetes is lack of effective insulin in the body	*21*	5
3. Diabetes is caused by failure of the kidneys to keep sugar out of the urine	2	*24*
4. Kidneys produce insulin	16	*10*
5. In untreated diabetes, the amount of sugar in the blood usually increases	*24*	2
6. If I am diabetic, my children have a higher chance of being diabetic	*10*	16
7. Diabetes can be cured	16	*10*
8. A fasting blood sugar level of 210 is too high	*7*	19
9. The best way to check my diabetes is by testing my urine	8	*18*
10. Regular exercise will increase the need for insulin or other diabetic medication	21	*5*
11. There are two main types of diabetes: Type 1 and Type 2	*8*	18
12. An insulin reaction is caused by too much food	8	*18*
13. Medication is more important than diet and exercise to control my diabetes	6	*20*
14. Diabetes often causes poor circulation	*24*	2
15. Cuts and abrasions on diabetes heal more slowly	*26*	0
16. Diabetics should take extra care when cutting their toenails	*26*	0
17. A person with diabetes should cleanse a cut with iodine and alcohol	9	*17*
18. The way I prepare my food is as important as the foods I eat	*25*	1
19. Diabetes can damage my kidneys	*22*	3
20. Diabetes can cause loss of feeling in my hands, fingers and feet	*25*	1
21. Shaking and sweating are signs of high blood sugar	22	*4*
22. Frequent urination and thirst are signs of low blood sugar	13	*13*
23. Tight elastic hose or socks are not bad for diabetics	19	*7*
24. A diabetic diet consists mostly of special foods	17	*9*

*Problem Areas in Diabetes* (PAID) [31] The 20 item PAID was measured with a five point Likert scale ranging from ‘Not a problem at all’ (1) to ‘A serious problem’ (5). High scores indicate that individuals are experiencing emotional distress in relation to living with diabetes. This instrument was altered by excluding Item no. 20, ‘Feeling burned out by the constant effort needed to manage diabetes.’ because it was not being well understood. This reduced the scale to 19 items (see Table 6).

**Table 6:** Results of Problem Areas in Diabetes

	*PAID* (n = 26) *m* = 4.2, SD 9.72, range min. 55–max. 95	
1.	I have no clear plan for the long term control of my diabetes	4.3
2.	I feel discouraged about the treatment of my diabetes	4.7
3.	I feel anxious when I think about living with my diabetes	4.7
4.	Unpleasant social situations can come from living with diabetes (for instance, others telling me what to eat)	4.0
5.	I have the feeling that I should miss food and meals	4.4
6.	I feel depressed when I think about living with diabetes	3.3
7.	I don’t know whether my mood depends on the level of my blood sugar	3.9
8.	I have the feeling that my diabetes is controlling my life	4.3
9.	I am worried about low blood sugar levels	4.2
10.	I am angry when I think about living with diabetes	3.4
11.	I am worried about food and meals all the time	4.1
12.	I am worried about the future and the risk of suffering from serious problems because of having diabetes	3.7
13.	I have the feeling that friends and family are not supporting my efforts to control my diabetes	4.5
14.	I feel guilty or worried when my diabetes gets out of control	3.5
15.	I can’t accept my diabetes	4.5
16.	I feel left alone with my diabetes	4.8
17.	I feel ‘burnt out’/tired because of my ongoing efforts in controlling my diabetes	4.5
18.	I can’t handle the problems caused by my diabetes	4.2
19.	I am not satisfied with my relationship with my doctor/my nurse who are treating my diabetes	4.7

*The Hopkins Scale Checklist*-*25* (HSC-25) [32], was used as a psychosocial screening measure with 10 items for symptoms of anxiety and 15 items for symptoms of depression. Scoring on each item of the two subscales was on a 4 point Likert scale ranging for ‘Not at all’ (1) to ‘Extremely’ (4). The cut-off point for the HSCL-25 was 1.75. A score above this indicates significant risk of anxiety and depression (see Table 7).

**Table 7:** Results of Hopkins Scale Checklist-25 (HCL-25)

	*Hopkins Scale Checklist*-*25 (n* = *26)* item cut off = 1.75	
	*Anxiety (10) mean* *=* *2.0 SD* *=* *0.3 range 1.5–2.3*	
1.	Suddenly scared for no reason	2.3
2.	Feeling fearful	2.3
3.	Faintness, dizziness, or weakness	1.7
4.	Nervousness or shakiness inside	1.7
5.	Heart pounding or racing	1.7
6.	Trembling	2.0
7.	Feeling tense or keyed up	1.8
8.	Headaches	1.5
9.	Spells of terror or panic	2.1
10.	Feeling restless, can’t sit still	2.3
	*Depression (15) mean* *=* *2.1 SD* *=* *0.5 Range 1.5–3.0*	
11.	Feeling low in energy, slowed down	1.5
12.	Blaming yourself for things	1.8
13.	Crying easily	2.3
14.	Loss of sexual interest or pleasure	1.6
15.	Poor appetite	2.1
16.	Difficulty falling asleep, staying asleep	1.5
17.	Feeling hopeless about the future	2.7
18.	Feeling blue	2.2
19.	Feeling lonely	2.4
20.	Thoughts of ending your life	2.8
21.	Feeling of being trapped or caught	2.2
22.	Worrying too much about things	1.9
23.	Feeling no interest in things	2.1
24.	Feeling everything is an effort	1.7
25.	Feelings of worthlessness	3.0

*Eye Health Questionnaire (EHQ*) The EHQ included questions relating to past eye health behaviour, whether individuals knew they needed to have annual eye screening and what barriers prevented them from attending for DR screening (see Table 1).

### Analysis

The psychometric data were double entered and analysed in Excel. The qualitative structured interviews were thematically coded using Atlas-ti.

## Results

### Demographic data

Among the 26 participants, age ranged from 48 to 104 years, although in this community knowledge of dates of birth are unreliable. Average number of children reported per family was 3 children. Occupation groups included: professionals (4), technicians (1), service workers (1), agricultural workers (4) and elementary workers (e.g. cleaners, labourers etc.) (3). Some occupations were not recorded because people were either self-employed as subsistence farmers, retired or unemployed (13) [37]. Only 3 members of the group had any secondary education, 10 members of the group had some primary education (standard 4–8), the rest did not give any indication of having received any education. The mean duration of diabetes was 8 years.

### Qualitative measures

Of the themes from the structured interviews with PLWD, 5 of the 9 themes demonstrated high to moderate levels of self-efficacy, as described in SCT: [15–17] these included seeking initial diagnosis; dialoguing with others about having diabetes (stigma); social support and raising public awareness; coping with sick days; seeking alternative therapy (herbalists), and seeking on going treatment. The remaining 4 themes demonstrated lower levels of self-efficacy: expression of emotion at diagnosis; engaging in dialogue about treatment; burden of disease and associated changes in life-style in terms of understanding healthy living related to diet & exercise

*High to moderate levels of self*-*efficacy*—When seeking the initial diagnosis more than half of the group initiated the consultation that resulted in their diagnosis of diabetes, exercising control over their actions, and demonstrating a high level of self-efficacy The remainder of the group followed the advice of family, friends or health care workers to seek a diagnosis for their symptoms.

Ref 27: 3 ‘I felt thirsty at night and drank a lot of water. Some people advised me to go and check for diabetes. Then I went to Kibongoto and they told me I had diabetes, about fifteen years ago’.

Self-efficacy was high for the majority of the group who were open about talking to family, friends and members of the community about diabetes for a number of reasons: for transport, for care, when socialising (to explain why they did not take sugar in their diet), telling others to raise awareness of diabetes.

Ref 29:32 ‘It is good to tell others because you might need their assistance and they ought to know where you are up to.’

Ref: 36: 19 ‘I am free (to talk to others about diabetes) and I am trying my best to tell people that they should check early. In case they have diabetes they can be advised on how to live.’

Coping with sick days showed moderately high self-efficacy. People used a number of different strategies to cope when they felt ill, one third reported that they took medication, a little under a third went to the hospital; to see the doctor or to have their blood glucose checked and their medication altered, some rested, took fluids and ate some food.

Ref 31: 31 ‘Í go to hospital to check whether the amount of sugar has gone up or lowered.’

Ref 27: 11 ‘I go to check and get advice whether to increase or reduce the amount of tablets I take.

The decision not to seek alternative therapy (herbalists) revealed high levels of self-efficacy. Some had never consulted a herbalist (13 out 20). By contrast 6 out of 20 people admitted to having consulted a herbalist but stated that the treatment was unsuccessful; At least one person admitted to being flexible about using a herbalist, although doctors had advised against this. Strong emotions were expressed about the negative implication of treatment by a herbalist;

Ref 34:29 ‘I can’t say that traditional healer can check me because they have nothing that they can use to tell me what the level of sugar in my body is. Their medicines too are not proven to be curing diabetes, so you just take it without knowing anything. So I can’t say that it helps treat diabetes.’

Ref 43: 38 ‘They have nothing…liars. They will make you grieved over your relatives and neighbours.’

The majority of people (24/27) were positive about seeking regular ongoing treatment demonstrating moderately high levels of self-efficacy. Only three people in the group admitted that they only went for a diabetic check-up when they felt ill. During treatment the relationship with health care-workers was described positively as a good or very good relationship. Health care workers were further described as kind, respectful and helpful and some were thankful for the care they received. (This was contrary to the findings in the PAID questionnaire, item *19*. *I am not satisfied with my relationship with my doctor/my nurse who are treating my diabetes (m* = *4.7).*

Specific barriers to on-going treatment were mentioned by six people, including: Not being able to get medication, having to buy one’s own medication at the private dispensary, the expense of diabetic care/management and one person commented on long waiting times at KCMC Hospital. Modes of transport to access on-going treatment included: bus (15) walking (9), bike, motorbike, taxi (1 each) with some people using more than one mode of transport. The time taken to reach the diabetic clinic from home varied from 5 to 10 min (2), ½ h (8), 1 h (2), 1½ h (3) to 2 h (3). The time taken to access regular treatment was not recorded for 8 participants.

*Lower levels of self*-*efficacy* were noted by a number of participants. The majority of people expressed negative emotions at the diagnosis of diabetes, ranging from anger, shock, sadness and sorrow to worry. One person was relieved with the diagnosis of diabetes, as prior to this diagnosis he thought that he was HIV positive and was going to die. Another person on learning that she had diabetes felt free from the anxiety of not knowing why she felt ill.

Ref: 30: 07 ‘…I have had many problems…One day…I was very thirsty and the muscles were extremely tight. I would get very tired while I was walking. I started losing weight everyday despite the excellent food cooked. I went to a doctor….I didn’t know the issues with diabetes. The doctor checked but did not tell me whether I had diabetes. I told my fellows that I was an HIV positive person. I was taken to a UN camp where I was checked out. The result came back to be HIV negative. I thought that maybe they were lying to me and that I would kill myself if they told me the truth. I stayed like that. All the time I was tired and weak. I was in Kibongoto. I could not even get up and my colleagues help me out. The day I found out that I had diabetes I urinated a lot and drank five litres of water. I came to find out that I had diabetes in 2003.

Ref 27: 34 ‘I feel free. Now that I know my disease, I simply feel free.’

Poor engagement in dialogue about treatment demonstrated low self-efficacy in self-management of this area of living with diabetes: When commenting on the service received at the hospital most described the routine activities of attending the diabetic clinic, being seen by the health care workers, having their blood sugar levels checked and medications dispensed. Only two people reported explaining things to the doctor and one reported asking for their blood sugar levels so they could give this result to family members. One person commented on being given advice.

Ref 45:13 ‘When I get there I provide money for consultancy and stay on queue until the time to be called comes. Then the doctor comes. Then the doctor would check and if there is a need for medicine they would provide.’

Ref 34: 26 ‘I ask the doctor how the level of my sugar in my body is so that I can tell the people at home my progress.’

Low self-efficacy was also demonstrated in the area of burden of disease People found that diabetes affected their quality of life, specifically in planning, organising and executing key activities of daily living. The ability to work, travel and socialise was mentioned by eight people. For twenty-two PLWD the physical impact of living with diabetes was mentioned including weakness, fatigue, reduced levels of energy, pain, reduced mobility, loss of independence and the effects of reduced eye sight. Some of the group were aware that following instructions about diet and medication was important and that not following this advice had negative consequences. This had resulted in a loss of personal freedom. They found this difficult and it made them feel unhappy.

Ref 45:24…’I don’t feel happy because I’m restricted to eat. I’m told not eat any red meat except for chicken and fish. I’m not very happy. I am allowed to eat all kinds of vegetables.’

Ref: 43: 9 ‘If you don’t continue with treatment properly, diabetes can affect you really badly due to the reasons I said earlier…’

Ref: 27:25…’changes to eating sweet things. So it was hard to eat what I was told to eat by the doctors.’

Coping with changes in life-style demonstrated the most striking area of low self-efficacy in this group: When asked, ‘Once you had diabetes, what changes did you make regarding your lifestyle about being diabetic?’ Change in diet appears to have been the most difficult and challenging aspect of life-style change facing this group. ‘We are told the food types…It is very hard to follow.’ The concept of life-style change and being able to eat a healthy diet in this area of self-management appeared not to have been comprehensively taught or understood by most of the group, ref 40:7.

Of the group 18 people specifically mentioned having to change their diet. Of these 11 people said that listening to what the doctor said about changing diet was important and 8 people mentioned that they had to eat what they had been instructed to eat (by the doctor).Reduction of carbohydrate intake was specifically mentioned (14) although only one person mentioned foods such as bread, rice and ugali (maize meal). Some culturally difficult issues were specifically mentioned: having to drink sugarless tea (6 people), increasing the intake of fruit and/or vegetable (18 people), the need to reduce the quantity of food eaten at each meal (3 people), and cook vegetables with less oil (2 people). Only two people mentioned the principle of eating a healthy diet.

Ref 27:10 ‘We are told the food types but it is hard to follow exactly what these types are. It is very hard to follow.’

Ref 42: 9 ‘If I don’t follow the instructions on food types, it can affect me severely. I don’t eat high sugar content food.’

Ref 40:7 ‘There are no specific food types for a diabetic patient but it is the quantity that matters. When you are eating you need to make sure that there are fruits, vegetables, protein and carbohydrates in order to have a complete meal.’

Ref 39:25 ‘There are many things that I have stopped after getting diabetes. For instance alcohol consumption, meat and high cholesterol content food…We are told to eat more vegetables and should not be added too much oil.’

Within the group there were more unusual ideas about specific food for a person living with diabetes:

Ref 34:22 ‘I was told to eat non carbohydrate foods.’

Ref 44:22 ‘I stopped eating a variety of food types. So I became thin due to that.… A little bit of bananas and barbeque meat.’

### Quantitative measures

#### Self-efficacy (Table 3)

The mean scores on the five subscales reflect high levels of self-efficacy in each area of self-care with the exception of Item 10 (nutrition/treatment *m* = 4.2) ‘I think that I’m able to keep to my diet most of the time’ (*m* = 3.3). This indicates low self-efficacy for this aspect of self-care. This is supported by findings in the structured interview.

#### Diabetic health beliefs (Table 4)

This psychometric measure is divided into five subscales. In the first subscale the majority of participants believed their diabetes was well controlled (*m* = *4.2).* Diet appears to be a burden to this group of PLWD. In the theme “Barriers (to diet and medication)” item 2, *I would have to change too many habits to follow my diet* (diabetic foods) (*m* = 3.9) is the highest perceived barrier to achieving this health behaviour. In the theme “Impact of Job on Therapy”, item 10*, If I changed “jobs” it would be easier to follow my diet (diabetic foods)* (*m* = 1.9) is the highest perceived barrier to achieving this health behaviour. Benefits of Therapy (diabetic foods, diabetic medication and weight control, mean scores for all 11 items of = 4.1 or more) and Social Support (for diet, *m* ≥ 2.4) are both perceived as being beneficial in maintaining health.

#### Diabetic knowledge questions (Table 5)

There were clear gaps in knowledge. Knowledge overall was poor (*m* = 15.92, 61.25 %); only 15 % were correct on knowledge on impact of exercise on need for medication (item 10) and only 19 % were correct about sign of hypoglycaemia (item 21).

#### PAID (Table 6)

On the PAID scale the mean was 4.2, SD 9.72 (range = 55–95), suggesting a high level of emotional distress overall amongst this group. Item 3, *I feel anxious when I think about living with my diabetes* (m = 4.7) is supported by the findings on the anxiety subscale of the HSCL-25 below and item 19 *I am not satisfied with my relationship with my doctor/my nurse who are treating my diabetes (m* = *4.7)* is an interesting contrast to the responses in the standard interview on the relation between PLWD and health care workers.

### Hopkins Scale Checklist-25 (Table 7)

On the anxiety subscale 6 out of the 10 items were above the 1.75 cut off suggesting high levels of anxiety among the group. Item 2. *Feeling fearful* (*m* = 2.3) would appear to reflect the state of anxiety on this subscale. This is in support of item 3 on the PAID scale above, *I feel anxious when I think about living with my diabetes* (*m* = 4.7)

On the depression scale 11 out of the 15 items were above the 1.75 cut off point, suggesting high levels of depression among the group, as are reflected by item 20. *Thoughts of ending your life* (*m* = 2.8) and item 25 *Feelings of worthlessness* (*m* = 3).

### Eye Health Questionnaire (EHQ)

All PLWD who responded to the request take part in the study were offered DR screening. A total of 79 people were screened for DR over the course of the NA, of these PLWD 29 were male and 50 were female. There was no difference in visual acuity between those included in the NA and the extra PLWD who were screened for DR. Visual acuity was recorded for 71/79 of all of those screened, and coded according to presenting distance visual acuity (see Table 8). Outcome of visual acuity assessment is shown in Table 9, and outcome DR findings are shown in Table 10. There were signs of DR in 9 of those whose were screened; dot blot haemorrhages, cotton wool spots, exudates. In one case there was also cataract in the other eye. Each person who was screened for DR was asked to complete the EHQ, the results are shown in Table 1. Reasons given for not previously being screened for DR included: cost—2 (2.5 %), ill health—2 (2.5 %), access to the clinic—3 (3.8 %), unaware of the need—23 (29.1 %), other reasons—19 (20 %). No reasons given—30 (38 %).

**Table 8:** Coding of presenting distance visual acuity (ICD revised changes to definition of blindness)

0 = Mild or no visual impairment: equal to or better than: 6/18
1 = Moderate visual impairment: worse than: 6/18/Equal to or better than: 6/60
2 = Severe visual impairment: worse than: 6/60/Equal to or better than: 3/60
3 = Blindness: worse than: 3/60/equal to or better than: 1/60
4 = Blindness: worse than: 1/60/equal to or better than light perception
5 = Blindness: no light perception
9 = Undetermined or unspecified

**Table 9:** Outcome of visual acuity coding for DR screening

CODES	0	1	2	3	4	5
Best eye	62	11	2	0	0	0
Worst eye	12	4	4	1	0	1

**Table 10:** Summary of findings from DR screening

Moderate visual impairment	Severe visual impairment	Diabetic retinopathy	Cataract
13.92 %	2.5 %	11.39 %	5.06 %

## Discussion

In much of Sub Saharan Africa services for detecting and treating DR are often rudimentary and largely confined to urban centres. Many barriers to the care of diabetic retinopathy in the region have been identified. In addition to the obvious shortage of ophthalmologists and their lack of training in DR, low numbers of other ancillary ophthalmic staff such as optometrists and poor training in retinal examination to detect DR compound the problem. Furthermore there are inadequate referral systems from primary to secondary care and from diabetic clinics to ophthalmic services. The latter are poorly resourced in terms of imaging equipment and treatment infrastructure such as lasers [38, 39].

However the needs of PLWD and how these might interact with the health services have not been studied.

### Health services

In the structured interview, PLWD spoke positively about their relationship with the health care workers caring for them, yet the PAID scale revealed a different picture. In answer to item 19 it was clear that there was dissatisfaction with relationship with those treating PLWD (*m* = 4.2). People were reluctant to openly criticise those who provided them with regular care or to talk about the limited resources that were clearly evident to all who visit the hospitals and clinics and are reported by the health care workers themselves. Given the lack of training in DM and chronic staff shortages in SSA [38] it is not surprising that PLWD revealed a level of dissatisfaction with health care workers.

### Education, knowledge

Burgess in his discussion on the emerging epidemic of diabetes in SSA calls for the education and empowerment of patients as an important part of disease management [39].

The assessment of diabetes knowledge with the DKQ [30] and the structured interview and the EHQ revealed gaps in knowledge and diabetes self-management, self-care and dietary advice among the group that needed to be addressed as part of a health intervention programme. The EHQ revealed that knowledge of need for eye screening and eye health services did not result in an uptake of eye health services. Knowledge does not lead to a change in health behaviour [10]. More has to be done to ensure that PLWD are screened annually for DR than making them aware that diabetes damages the eye. SCT suggests knowing what to do must be supported by the cognitive, social, emotional and behavioural skills to adopt, maintain and persist with health behaviours in the face of difficulties and obstacles [17]. The gap between knowledge and behaviour helps explain why one of the main challenges faced by new screening services has been uptake of referrals to the eye department after a positive screening test [11].

### Uptake of screening for DR

Previous research conducted on the uptake of eye health service in Kilimanjaro show many barriers exist [40]. These include socio-cultural challenges; elderly people may have to ask relatives for social and financial support when they need eye treatment and surgery. For some this is difficult when limited, seasonal resources are required for food, clothing and school fees. Ill health is defined locally as pain, so in many instances a loss of visual acuity does not meet this definition [40].

Our previous experience with DR screening in Kilimanjaro showed the positive effect of providing patient education and counselling in the diabetic clinic. Having a nurse counsellor present in the diabetic clinic at KCMC Hospital increased the uptake of screening for DR from 29 to 47 % [9]. However the fact that more than 50 % of patients did not take advantage of screening services in the same hospital could be explained by some of the findings of our needs assessment study that relate to self-efficacy, anxiety and depression.

### Low self-efficacy

For most of the group the diagnosis of diabetes demonstrated low levels of SE. Dietary change was the area of the lowest self-efficacy, compounded by minimal dialogue between PLWD and health care workers who appeared to give instructions on life style changes without explanations that would lead to understanding, and in turn raise levels of self-efficacy. Low SE was clearly expressed when people were asked how diabetes had affected their lives. One person felt that diabetes had left her bereft of family, (ref 45:4) there was loss of employment and income, loss of mobility, a loss of vision and independence with an associated reliance on others. All of these affect people’s relationships with their families, their friends and their sense of self-worth. Low SE as part of engaging in dialogue about treatment may well be both a cultural phenomenon and the paternalistic approach to health care in Tanzania [41]. It may contribute to a large portion of those who did not attend for screening in our original study and needs to be addressed when planning DR screening services.

### Anxiety and depression

It is telling that as a measure of emotional distress the highest scoring item on the PAID scale [31] was feeling anxious about living with diabetes (items 3 = 4.7). This triangulated with findings on both the HSCL-25 and the structured interview. The emotional burden of living with diabetes cannot be under estimated especially in communities were resources are limited and poverty is also a factor. There is a need for comprehensive mental health care to be integrated into diabetes care [42].

### Stigma

Diabetes related stigma has received little attention worldwide [43] and none in Africa. Type two diabetics in Australia felt discriminated against with fewer opportunities in life, subject to negative stereotyping and felt blamed by others for their condition [44]. On the other hand type one diabetics did not experience similar social stigmatisation.

The group of PLWD in Tanzania were type two diabetics. In contrast to the Australian group of diabetics, the Tanzanian group was highly motivated to tell others about diabetes in order to raise community awareness about diabetes to prevent others suffering from diabetes without treatment and to ensure they received support for themselves for their on-going treatment and care. This shows a lack of stigma towards diabetes amongst this group. Following this initial NA patients being screened for DR were given a Kilimanjaro Diabetic Programme (KDP) sticker to place on their blouse, dress or shirt to indicate that they had received screening. The aim was to promote dialogue in the community about screening. Two things were observed about these stickers. Firstly, patients who had undergone screening continued to return to KCMC Hospital week after week still wearing their stickers. If the staff who conducted the DR screening forgot to give a sticker to a patient after screening, the patient would return and ask for a sticker. It seemed that amongst this group of PLWD that there was no stigma of being diabetic and the object of giving out the KDP stickers to raise awareness in the community by having people wear them worked. In a continent with a high prevalence of a more stigmatising disease such as HIV/AIDS, it may be a relief to PLWD to have a more socially acceptable explanation for their chronic illness.

## Conclusions

This needs assessment reveals dissatisfaction with the health workers treating their condition and low self-efficacy in areas of managing their diabetes and preventing complications, as well as a lack of knowledge about DM and its complications. High levels of anxiety and depression are experienced by PLWD. On a positive note there seems to be little stigma attached to a diagnosis of DM. These insights are useful in planning diabetic retinopathy screening and treatment services.

The KDP has moved through 4 of the 5 phases of implementing a DR screening service as described by Poore et al. [45]. These start with screening in the eye clinic, move to screening in the hospital diabetic clinic, create a diabetes register and call for eye screening and identifying undiagnosed DR in the community.

The quantitative measures and qualitative interviews, together with the findings of the EHQ for PLWD and a NA conducted with health care workers in Kilimanjaro Region were of value in showing the need for the comprehensive health intervention programme that was subsequently developed by the KDP [11]. The KDP was established with the combined efforts of government representatives, hospital directors, epidemiologists, endocrinologists ophthalmologists and social scientists [11]. Clearly there was a need for both diabetes education and eye health services at district level in Kilimanjaro region. The KDP has taken diabetes education to health care providers in Kilimanjaro region at district level so that they can provide education to PLWD on all aspects of self-care and management of living with diabetes.

As was seen from the EHQ, knowledge that DM damages the eye did not mean that PLWD would go for DR screening. The KDP developed a diabetic diary that allows PLWD to record their regular clinic visits and review their blood pressure, blood glucose, and weight. The diary also contains useful information for patients including sections on diet, exercise, foot care and eye health. The KDP facilitators subsequently led 3 years of annual educational workshops at district level in Kilimanjaro region. Two cartoons on DR were developed to provide patient information to PLWD when they attended for DR screening.

The DR screening programme is supported by an electronic database. The KDP electronic database of all known diabetics in Kilimanjaro was set up so that all PLWD, in Kilimanjaro region, can be registered and their clinic, and hospital attendance, screening and treatment for DR are electronically recorded. This allows for recording and review of retinal photographs and reminders to be sent automatically for review and treatment.

By the beginning of 2015, 3463 (60·4 %) of the 5729 individuals registered with the KDP, had been screened for retinopathy. The prevalence of any diabetic retinopathy was 27·9 % in those screened. Proliferative retinopathy was present in 2·9 % and maculopathy in 16·1 % of individuals who were screened [11, 12].

The KDP aimed to make screening for DR more accessible to PLWD by taking DR screening to the district level. The KDP helped to establish dedicated diabetes clinics in 18 peripheral hospitals, with the aim of providing knowledge based teaching, clinical support, resources and action plans that health care workers could use to empower PLWD. The mobile diabetic retinopathy screening team work through these centres.

## Limitations and strengths

We conducted a large number of interviews and believe that this constitutes a reasonable representation of the views of people living with diabetes in the Kilimanjaro Region. However the subjects were not randomly recruited and there is the possibility of bias with for example subjects with a higher level of self-efficacy in managing their diabetes presenting for the needs assessment interviews.

## Abbreviations

DR: diabetic retinopathy
SE: self-efficacy
DKQ: Diabetes Knowledge Questionnaire
DHB: Diabetes Health Beliefs
PAID: Problem Areas in Diabetes
HCL-25: Hopkins Scale Checklist-25
DM: Diabetes mellitus
PLWD: people living with diabetes
NA: needs assessment
KDP: Kilimanjaro Diabetic Programme
EHQ: Eye Health Questionnaire
SSA: Sub-Saharan Africa
